# NSABP FB-7: a phase II randomized neoadjuvant trial with paclitaxel + trastuzumab and/or neratinib followed by chemotherapy and postoperative trastuzumab in HER2^+^ breast cancer

**DOI:** 10.1186/s13058-019-1196-y

**Published:** 2019-12-03

**Authors:** Samuel A. Jacobs, André Robidoux, Jame Abraham, José Manuel Pérez-Garcia, Nicla La Verde, James M. Orcutt, Marina E. Cazzaniga, Fanny Piette, Silvia Antolín, Elena Aguirre, Javier Cortes, Antonio Llombart-Cussac, Serena Di Cosimo, Rim S. Kim, Huichen Feng, Corey Lipchik, Peter C. Lucas, Ashok Srinivasan, Ying Wang, Nan Song, Patrick G. Gavin, April D. Balousek, Soonmyung Paik, Carmen J. Allegra, Norman Wolmark, Katherine L. Pogue-Geile

**Affiliations:** 1 NSABP Foundation, Inc., Nova Tower 2, Two Allegheny Center – Ste 1200, Pittsburgh, PA 15212 USA; 2 Centre hospitalier de l’université de Montréal, Montréal, QC Canada; 3 Cleveland Clinic, Cleveland, OH USA; 4 QuironSalud Group, IOB Institute of Oncology, Madrid, Barcelona Spain; 5 Medica Scientia Innovation Research (MedSIR), Barcelona, Spain; 6 Present address: ASST Fatebenefratelli Sacco - PO Luigi Sacco, Milan, Italy; 7 ASST Fatebenefratelli Sacco - PO Fatebenefratelli, Milan, Italy; 8 Roper St. Francis Healthcare, Charleston, SC USA; 9 Azienda Ospedaliera San Gerardo, Monza, Italy; 10 International Drug Development Institute (IDDI), Louvain-la-Neuve, Belgium; 11 Hospital Universitario, Coruña, Spain; 12 Fondazione IRCCS Istituto Nazionale di Tumori, Milan, Italy; 13 Department of Pathology, University of Pittsburgh School of Medicine, Pittsburgh, PA USA; 14 Severance Biomedical Science Institute and Department of Medical Oncology, Yonsei University College of Medicine, Seoul, Republic of South Korea; 15 Department of Medicine, University of Florida Health, Gainsville, FL USA; 16 University of Pittsburgh, Pittsburgh, PA 15212 USA

**Keywords:** Breast cancer, Neoadjuvant, Neratinib

## Abstract

**Purpose:**

The primary aim of NSABP FB-7 was to determine the pathologic complete response (pCR) rate in locally advanced HER2-positive (HER2^+^) breast cancer patients treated with neoadjuvant trastuzumab or neratinib or the combination and weekly paclitaxel followed by standard doxorubicin plus cyclophosphamide. The secondary aims include biomarker analyses.

**Experimental design:**

pCR was tested for association with treatment, gene expression, and a single nucleotide polymorphism (SNP) in the Fc fragment of the IgG receptor IIIa-158V/F (FCGR3A). Pre-treatment biopsies and residual tumors were also compared to identify molecular changes.

**Results:**

The numerical pCR rate in the trastuzumab plus neratinib arm (50% [95%CI 34–66%]) was greater than that for single-targeted therapies with trastuzumab (38% [95%CI 24–54]) or neratinib (33% [95%CI 20–50]) in the overall cohort but was not statistically significant. Hormone receptor-negative (HR^−^) tumors had a higher pCR rate than HR^+^ tumors in all three treatment arms, with the highest pCR rate in the combination arm. Diarrhea was the most frequent adverse event and occurred in virtually all patients who received neratinib-based therapy. Grade 3 diarrhea was reported in 31% of patients; there were no grade 4 events. Our 8-gene signature, previously validated for trastuzumab benefit in two different clinical trials in the *adjuvant* setting, was correlated with pCR across all arms of NSABP FB-7. Specifically, patients *predicted* to receive no trastuzumab benefit had a significantly lower pCR rate than did patients *predicted* to receive the most benefit (*P* = 0.03). FCGR genotyping showed that patients who were homozygous for the Fc low-binding phenylalanine (F) allele for *FCGR3A-158V/F* were less likely to achieve pCR.

**Conclusions:**

Combining trastuzumab plus neratinib with paclitaxel increased the absolute pCR rate in the overall cohort and in HR^−^ patients. The 8-gene signature, which is validated for predicting trastuzumab benefit in the adjuvant setting, was associated with pCR in the neoadjuvant setting, but remains to be validated as a predictive marker in a larger neoadjuvant clinical trial. HR status, and the *FCGR3A-158V/F* genotype, also warrant further investigation to identify HER2^+^ patients who may benefit from additional anti-HER2 therapies beyond trastuzumab. All of these markers will require further validation in the neoadjuvant setting.

**Trials registration:**

ClinicalTrials.gov, NCT01008150. Retrospectively registered on October 5, 2010.

## Introduction

Human epidermal growth factor receptor 2 (HER2/*ERBB2*) is overexpressed in approximately 25% of breast cancers and drives hyperactivation of the HER2 pathway via downstream signaling initiated by receptor homo- or hetero-dimerization with other HER family members (HER2/HER2, HER2/EGFR, HER2/HER3). Prior to the advent of HER2-targeted therapeutics, HER2-positive disease (HER2^+^) was associated with a poor clinical outcome, but this dramatically changed with the development of trastuzumab and additional HER2-specific agents, especially in early HER2^+^ breast cancer [1–5]. Unfortunately, the vast majority of patients with metastatic HER2^+^ disease who initially respond to trastuzumab will develop resistance [6]. This has stimulated substantial interest in understanding both the mechanisms of resistance and development of additional anti-HER2 agents.

Small-molecule tyrosine kinase inhibitors (TKIs) suppress the growth of HER2^+^ breast cancer cells in vitro and tumor xenografts in vivo by inhibiting phosphorylation of *ERBB* receptor tyrosine kinases (RTKs) and thus preventing activation of downstream signaling mediators [7–9]. Lapatinib, a reversible inhibitor of EGFR and HER2 RTKs, is active in the first and subsequent lines of treatment of patients with HER2^+^ metastatic disease and has received US Food and Drug Administration (FDA) approval when combined with capecitabine in patients with progressive disease after anthracycline, a taxane, and trastuzumab [10]. In two neoadjuvant trials, NeoALTTO (*N* = 455) [11] and CALGB 40601 (*N* = 305) [12], the combination of lapatinib, trastuzumab, and paclitaxel was compared to paclitaxel combined with either anti-HER2 agent alone. The rate of pathologic complete response (pCR), defined as no residual invasive disease (in the breast), was numerically higher in patients receiving lapatinib and trastuzumab than in patients receiving trastuzumab alone (NeoALTTO 51% vs 29%, *P* = 0.0001; CALGB 56% vs 46%, *P* = 0.13). In both studies, a statistically significant increase in pCR was observed in patients with HR^−^ disease treated with dual anti-HER2 therapy compared to single-agent anti-HER2 therapy.

Neratinib, an irreversible inhibitor of pan-*ERBB* RTKs, is more potent than lapatinib in HER2^+^ breast cancer cell lines and in human tumor xenografts [13]. In phase II studies with neratinib monotherapy in patients with HER2^+^ breast cancer, the objective response rate was 24% in trastuzumab-refractory patients and 56% in trastuzumab-naive patients [14].

Clinical data in HER2^+^ metastatic breast cancer patients treated with neratinib at 240 mg/day and paclitaxel at 80 mg/m^2^ on days 1, 8, and 15 of a 28-day cycle resulted in a median progression-free survival of 47.9 weeks and an objective response rate of 71% with responses observed in patients with prior trastuzumab, lapatinib, and taxane therapy [15]. The combination of weekly paclitaxel with trastuzumab plus neratinib in women with metastatic, HER2^+^ advanced breast cancer was evaluated in the phase Ib NSABP FB-8 study (NCT01423123). The recommended phase II dose of neratinib was 200 mg/day. Impressive clinical activity was observed in these heavily pre-treated patients with an objective response rate of 38% and a clinical benefit rate of 52% [16].

Taken together, these studies suggest that combining non-cross resistant anti-HER2 therapy may result in a higher pCR rate. Here, we report the safety and efficacy of a randomized study in patients with locally advanced, HER2^+^ breast cancer treated in the neoadjuvant setting with trastuzumab, neratinib, or the combination, in each case administered for 16 weeks with paclitaxel followed by standard chemotherapy with doxorubicin plus cyclophosphamide (AC) for 4 cycles.

## Methods

### Patients and study design

This phase II trial, which opened for accrual in the US and Canadian NSABP Foundation sites, was designed originally as a two-arm study with 2:1 randomization to evaluate trastuzumab or neratinib with paclitaxel followed by AC. In December 2011, after 30 patients were enrolled, accrual was placed on hold. The decision to hold accrual was based on reports that dual anti-HER2 inhibition with trastuzumab plus lapatinib or trastuzumab plus pertuzumab increased the pCR rate in neoadjuvant breast cancer [11, 17]. From May 2011 to July 2012, the NSABP Foundation conducted a phase I dose-escalation study in patients with HER2^+^ metastatic disease evaluating the combination of trastuzumab, neratinib, and paclitaxel. This study established the recommended phase II dose of neratinib as 200 mg/day in combination with trastuzumab and paclitaxel [16]. In August 2012, FB-7 reopened as a randomized (1:1:1) study adding the third arm of trastuzumab, neratinib, and paclitaxel followed by AC. The randomization was adjusted so that at the completion of the study, the three arms, would have an equal number of evaluable patients (total study *N* = 126). On September 30, 2013, the US FDA granted accelerated approval to pertuzumab in combination with trastuzumab and docetaxel for use in the neoadjuvant setting in women with HER2^+^ breast cancer based on improved pCR in the NeoSphere study (pertuzumab, trastuzumab, docetaxel, pCR rate 45.8%) [17] and TRYPHAENA study (pertuzumab, trastuzumab, carboplatin, docetaxel, pCR rate 66.2%) [18].

On October 22, 2013, the NSABP closed arms 1 and 2 of FB-7 to additional accrual in the USA. From October 22, 2013, to April 2014, arm 3 remained open as a non-randomized arm to accrue an additional 12 patients in order to obtain more robust safety information on US patients. These patients are included only in the biomarker analysis with the exception of the MammaPrint**®** analysis, which included only randomized patients. European sites, in which dual anti-HER2 therapy was not the standard of care, were opened to complete the accrual to the randomized three-arm trial (total study *N* = 126). Canadian sites continued to accrue to the three-arm trial. The last patient enrolled on November 12, 2014.

Eligible patients included women ≥ 18 years with ECOG PS of 0–1, invasive adenocarcinoma of the breast, HR^+^ or HR^−^ disease, and HER2 positivity defined as a score of 3+ by immunohistochemistry (IHC), or evidence of gene amplification by fluorescence in situ hybridization (FISH) or chromogenic in situ hybridization (CISH). HER2 testing was performed locally without central confirmation. We are not able to provide HER2 status based upon the guidelines published in 2018 [19].

Patients with AJCC stage IIB, IIIA, IIIB, or IIIC were eligible. Patients were required to have the following baseline laboratory studies: an absolute neutrophil count of ≥ 1000/mm^3^, platelet count of ≥ 100,000/mm^3^, hemoglobin of ≥ 9 g/dL, serum creatinine ≤ 1.5× the upper limits of normal (ULN), total bilirubin of ≤ 1.5× the ULN, AST, and ALT of ≤ 1.5× ULN. Left ventricular ejection fraction (LVEF) ≥ 50% assessed by either 2D echocardiogram or MUGA scan was required. The first 60 patients were required to have a research biopsy before therapy initiation; this was subsequently dropped as a requirement.

Patients were excluded if they had any evidence of metastatic disease, active hepatitis B or C with abnormal liver function tests, intrinsic lung disease causing dyspnea, persistent ≥ grade 2 diarrhea, sensory-motor neuropathy ≥ grade 2, conditions that would prohibit intermittent administration of corticosteroids for paclitaxel premedication, or active cardiac disease including recent myocardial infarction, symptomatic arrhythmia, or angina pectoris.

The study protocol was approved by the Institutional Review Boards of each participating institution, and all patients provided written informed consent. The study was conducted according to the Good Clinical Practice and the Declaration of Helsinki and its amendments. The authors had full control of all primary data. The datasets during and/or analyzed during the current study will be available from the corresponding author on reasonable request and with permission from PUMA Biotechnology.

Patients in arm 1 (control) received 4 cycles of paclitaxel 80 mg/m^2^ administered on days 1, 8, and 15 of a 28-day cycle with trastuzumab 4 mg/kg loading dose, then 2 mg/kg weekly for a total of 16 doses. Following paclitaxel and trastuzumab, doxorubicin (A) 60 mg/m^2^ and cyclophosphamide (C) 600 mg/m^2^ were given every 3 weeks for 4 cycles. In arm 2 (experimental), in place of trastuzumab, patients received neratinib 240 mg taken orally once daily beginning on day 1 of paclitaxel and continuing through day 28 of the final cycle of paclitaxel. In arm 3 (experimental), both trastuzumab and neratinib were given with paclitaxel as described above; however, neratinib was administered at 200 mg/day. After recovery from all chemotherapy, patients had their definitive surgery and completion of 1 year of trastuzumab. The decisions regarding hormonal therapy and radiotherapy were at the discretion of the treating physician.

Standard pre-medications were given before each paclitaxel administration and during AC. Because diarrhea is expected with neratinib, early in the study, diarrhea management was initiated after the first diarrheal stool with loperamide 4 mg and then 2 mg after each loose stool thereafter. Despite this management plan, diarrhea was still consistently noted within 2 weeks of study therapy. Subsequently, diarrheal guidelines were amended to mandate primary prophylaxis with loperamide beginning with the first dose of therapy. At all treating sites, patients were contacted at 24, 48, and 72 h during the first week of treatment on the neratinib-containing arms to reenforce anti-diarrheal management.

The primary endpoint was pCR, defined as no residual invasive disease in the breast and lymph nodes (*y*pT0/N0). Randomly assigned patients who received any protocol therapy were included in the analysis. Secondary endpoints included clinical complete response, pCR in the breast, and rate of adverse events (AEs). Due to the small sample size and a limited number of tissue samples available, all molecular correlates are exploratory.

### Study assessments

Safety was assessed by physical examination, interim history, and laboratory assessment. AE assessment occurred on days 1 and 15 of cycle 1 and on day 1 of each subsequent cycle, and 2 to 4 weeks after the last cycle of AC. AE reporting was assessed according to the National Cancer Institute Common Terminology Criteria for Adverse Events (NCI CTCAE) version 3.0. AEs occurring at the US or Canadian sites were continuously monitored and reviewed by the NSABP medical review team. European sites were monitored by MedSIR every 6 to 10 weeks.

### Molecular analyses

#### Tumor RNA extraction

RNA was isolated from pre-treatment tumor biopsies and residual disease utilizing one to five 5-μm tissue sections depending on the size of the tumor area within a slide. Separate tissue sections were required for RNA and DNA. Extractions were prepared using the AllPrep DNA/RNA FFPE kit from Qiagen and following the manufacturer’s recommendations.

#### *FCGR* genotyping

Whole blood was collected in ACD tubes from patients (*n* = 80) before treatment. Peripheral blood monocytes (PBMC) were isolated and stored at − 80 °C. Genotyping of rs1801274 (*FCGR2A-*131R/H) and rs396991 (*FCGR3A-*158V/F) was performed as previously described [20].

#### RNA-Seq

Details of the preparation, performance, and analysis of the RNA-Seq libraries are included in Additional file 1: Methods.

#### MammaPrint scoring

The proprietary MammaPrint 70-gene Breast Cancer Recurrence Assay was performed at Agendia, using 50 ng of RNA for all available patients with pre-treatment biopsies with sufficient RNA (*n* = 45); one case failed RNA and hybridization quality control, and in five cases, pCR information was missing. MammaPrint was performed retrospectively to determine the number of low-risk patients enrolled in the study.

#### Statistical analysis

This is a non-comparative, randomized phase II study in which patients with HER2^+^, locally advanced disease were randomized to one of the three arms. The study was designed originally as a two-arm trial with 2:1 randomization. However, when it became apparent that dual anti-HER2 regimens showed early favorable results, the trastuzumab plus neratinib arm was added and the randomization balanced among arms. The primary endpoint was pCR, defined as no evidence of invasive disease in the breast and nodes (*y*pT0/N0). We determined that a sample size per arm of 42 patients would offer 80% power to test the null hypothesis that the response rate would be 30% or less at a one-sided alpha of 0.05. pCR was analyzed descriptively by treatment arm and, for exploratory purposes, was compared across the three arms (two-by-two comparisons) using the stratified Cochran-Manel-Haenszel statistic (two-sided *P* values). The analyses were stratified in such a way that patients randomly assigned to the study prior to the addition of arm 3 were considered in a separate stratum from those randomly assigned after the addition of arm 3.

Secondary aims and exploratory molecular analyses were performed for exploratory purposes with Fisher’s exact or chi-square tests, not adjusted for multiplicity. Any comparison that reached nominal statistical significance should be interpreted with due regard to the multiple comparisons performed, the small number of patients in the subgroups, and the fact that some analyses were performed retrospectively.

## Results

### Patient characteristics

**Fig. 1:**
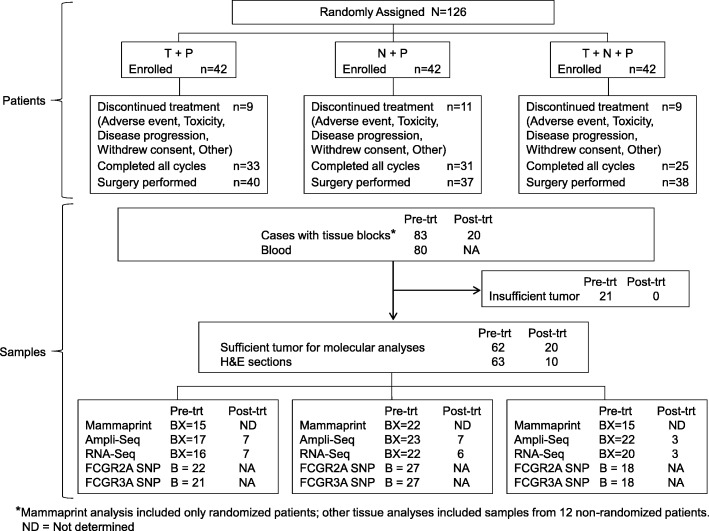
CONSORT Diagram: NSABP FB-7 study

There were 126 patients enrolled in the randomized portion of this study who received at least one dose of study drug and are included in Table 1. Distribution of tumor stage was similar across arms, as were tumor size and method of determination of HER2 status. There were more postmenopausal patients in arm 2 and fewer HR^+^ patients in arm 3 although this difference was not statistically significant. Three patients withdrew from the study before receiving any study drug and were replaced (Fig. 1 CONSORT Diagram: NSABP FB-7).

**Table 1:** NSABP FB-7: patient characteristics by randomized arm

	Arm 1, *n* = 42	Arm 2, *n* = 42	Arm 3, *n* = 42
Age, years			
Median	50.0	56.0	50.0
Range	33–71	29–71	31–77
Performance status			
ECOG 0	42 (100%)	42 (100%)	41 (97.6%)
ECOG 1	0	0	1 (2.4%)
Tumor stage			
IIB	18 (42.8%)	19 (45.2%)	20 (47.6%)
III	21 (50%)	20 (47.6%)	22 (52.3%)
Missing	3 (7.1%)	3 (7.1%)	0
Inflammatory	4(9.5%)	7(16.7%)	4(9.5%)
Menopausal status			
Pre	25 (60%)	16 (38%)	23 (55%)
Post	17 (40%)	26 (62%)	19 (45%)
Hormone receptor			
Positive	28 (67%)	29 (69%)	23 (55%)
Negative	14 (33%)	13 (31%)	19 (45%)
Clinical tumor size			
Mean, cm	4.50	5.04	4.72
Median, cm	3.70	5.50	5.00
Range, cm	0.0–18.0	0.0–10.0	0.0–9.0
HER2			
IHC	24 (57.1%)	24 (57.1%)	25 (59.5%)
ISH	15 (35.7%)	12 (28.6%)	11 (26.2%)
IHC and ISH	3 (7.1%)	6 (14.3%)	6 (14.3%)

### Adverse events

The most common grade 1–4 treatment-emergent AEs that occurred with anti-HER2 therapy plus paclitaxel are shown in Table 2. Diarrhea was the most frequent AE and occurred in virtually all patients who received neratinib-based therapy, with grade 3 diarrhea reported in 31% of patients; there were no grade 4 diarrheal events. During the course of the study, primary anti-diarrheal prophylaxis was instituted and resulted in a decrease in grade 3 diarrhea in the neratinib arms to 21%. Grade 2 constipation occurred in 5% and 2% of arms 2 and 3, respectively. Higher grades of constipation were not observed. Grade 3 transaminase elevation was observed in 7% of the patients in the neratinib-containing arms (Table 2), which is very similar to the findings in I-SPY2 [21].

**Table 2:** NSABP FB-7: treatment-emergent adverse events during trastuzumab plus paclitaxel (arm 1), neratinib plus paclitaxel (arm 2), or trastuzumab plus neratinib plus paclitaxel (arm 3) (all cycles)

Event	Arm 1, *n* = 42			Arm 2, *n* = 42			Arm 3, *n* = 42		
	Grades 1–2	Grade 3	Grade 4	Grades 1–2	Grade 3	Grade 4	Grades 1–2	Grade 3	Grade 4
Diarrhea	16 (38%)	0	0	29 (69%)	13 (31%)	0	28 (66%)	13 (31%)	0
Nausea	12 (29%)	0	0	25 (60%)	0	0	19 (46%)	1 (2%)	0
Rash	9 (21%)	0	0	9 (21%)	0	0	6 (14%)	0	0
Transaminase elevation	9 (21%)	1 (2%)	0	16 (38%)	3 (7%)	0	18 (43%)	3 (7%)	0
Fatigue	18 (43%)	0	0	21 (50%)	1 (2%)	0	17 (40%)	1 (2%)	0
Neuropathy	15 (36%)	0	0	13 (31%)	0	0	6 (14%)	1 (2%)	0

At least one neratinib dose reduction occurred in 38% of patients in arm 2 and 52% of patients in arm 3. During paclitaxel plus anti-HER2 therapy, at least one drug was discontinued before completion of 4 cycles in 19%, 21%, and 40% in arms 1, 2, and 3, respectively. Dose intensity of the targeted agents calculated until the time of discontinuation is shown in Additional file 1: Table S1.

### Efficacy and compliance

Clinical assessment in patients with a baseline palpable breast lesion reported an overall complete response in 70% of patients with 66%, 71%, and 74% in arms 1, 2, and 3, respectively. A total of 91% of patients had a definitive surgery equally distributed across arms. The pCR rates (the breast and nodes) were 38%, 33%, and 50% in arms 1, 2, and 3, respectively (see Table 3). pCR rates in the breast were 50%, 38%, and 52% in arms 1, 2, and 3, respectively. There were 11 patients for whom we do not have surgical findings. Reasons include early progression (*n* = 3), consent withdrawal, or lost to follow-up (*n* = 8). All patients for whom data are missing were considered no pCR. At 2 years from the random assignment, recurrence had been reported in five patients (arm 1 = 0, arm 2 = 3, and arm 3 = 2). No deaths were reported.

**Table 3:** NSABP FB-7: pathologic complete response (pCR) (breast and nodes) by treatment arm and HR status

Treatment arms, *n* = 42 each arm	Total pCR	*P*-value for pCR T vs Other Arms in all patients^a^	pCR HR^+^	P-value for pCR T vs Other Arms T in HR^+b^	pCR HR^**−**^	P-value for pCR T vs Other Arms T in HR^**−**b^	*P* HR^+^ vs HR^**−**^
Arm 1 (T)	16/41 (39.0%)	NA	8/27 (29.6%)	NA	8/14 (57.1%)	NA	0.11
Arm 2 (N)	14/42 (33.3%)	0.63	8/29 (27.6%)	1	6/13 (46.2%)	0.71	0.30
Arm 3 (T plus N)	21/42 (50%)	0.22	7/23 (30.4%)	1	14/19 (73.7%)	0.46	0.01
All arms	51/126 (40%)		23/79 (29%)		28/46 (61%)		0.001

*T* trastuzumab, *N* neratinib

^a^CMH test stratified for the time period the patients were randomized to the study (prior/after the addition of arm 3)

^b^Fisher’s exact test

### Assessment of breast cancer recurrence risk

The I-SPY2 trial (19) excluded patients with low risk recurrence score based on the MammaPrint 70-gene breast cancer recurrence assay.

In FB-7, we did not use this assay to prospectively exclude low-risk patients, although we retrospectively assessed the risk of recurrence based on the MammaPrint score in 45 randomly assigned patients. Analysis of the available samples showed that there were only 3 patients (5.8%) with a low-risk MammaPrint result (Additional file 1: Figure S1). No patient with a low-risk MammaPrint score had a pCR. There were 2 patients with low-risk disease in the trastuzumab arm and 1 in the neratinib arm. Thus, it is unlikely that the inclusion of patients at low risk was responsible for the discordant results between I-SPY2, which showed single-agent neratinib to be superior to trastuzumab in HER2^+^ and HR^−^ patients, whereas FB-7 showed no difference in pCR rates between single-agent neratinib and trastuzumab.

### Hormone receptor status and pCR

We tested the association of HR status with pCR (*N* = 126). Similar to other neoadjuvant studies (Additional file 1: Table S2), patients with HR^+^ tumors were less likely to achieve pCR after treatment with anti-HER2 therapies than those patients who were HR^−^ (Table 3). This difference in pCR rates between HR^+^ and HR^−^ patients reached statistical significance only within arm 3 (trastuzumab + neratinib + paclitaxel) (Table 3). HR^−^ patients who were treated with the trastuzumab plus neratinib combination had the highest numerical pCR rate (73.7%), although this rate was not statistically different compared to HR^−^ patients treated with trastuzumab (57.1%, *P* = 0.46).

### *FCGR* genotyping

Antibody dependent cellular cytotoxicity (ADCC) is a mechanism to determine the therapeutic efficacy of monoclonal antibodies such as trastuzumab. ADCC is initiated when the Fcɣ receptor IIIa (FcɣRIIIa) on NK cells binds to the Fc region of trastuzumab. FcɣIIIaR has been shown to have a differential affinity to the Fc portion of IgG1 depending upon a SNP at position 158 (V/F) in the *FCGR3A* gene. The FcɣRIIIa-158V protein has a higher affinity for IgG1 antibodies than does the FcɣRIIIa-158F protein. We have previously shown an association of the *FCGR3A-158F/V* genotypes with benefit from trastuzumab in the adjuvant setting [20]. FB-7 provided an opportunity to test for an association of the *FCGR3A-158F/V* genotypes with pCR in the neoadjuvant setting. The pCR rate for patients with an *FCGR3A* genotype with one or two of the high-binding alleles (*FCGRA-158V*) was 19/31 [61.3%], in comparison with only 7/27 [25.9%] for patients who were homozygous for the low-binding allele (*FCGRA-158F*). The difference in pCR rates between low- (F/F) and high-binding alleles (F/V or V/V) was statistically significant for all patients analyzed together (*P* = 0.009) and also for patients in the trastuzumab arm (*P* = 0.02), but not in the neratinib (*P* = 0.4) or in the trastuzumab plus neratinib arms (*P* = 0.6), which would be predicted based on the mechanism of action of neratinib (Table 4). Because the therapeutic efficacy of neratinib is not dependent upon eliciting antibody-dependent cellular cytotoxicity (ADCC) activity, we postulated that patients with the *FCGR3A-158F/F* genotype would have a better pCR rate in the trastuzumab plus neratinib arm than in the trastuzumab arm [1]. Indeed, we do see a numerically higher pCR rate in the trastuzumab plus neratinib arm (3/7 = 43%) than in the trastuzumab arm (1/8 = 13%).

**Table 4:** NSABP FB-7: association of *FCGR3A-158F/V* single nucleotide polymorphisms (SNP) with pathologic complete response (pCR)

Treatment arm	pCR status	F/F patients	pCR rate in F/F (%)	V/V or V/F patients	pCR rate in V/V or V/F (%)	Compare F/F to V/F and V/F *P* values
Trastuzumab	No pCR	7	13	3	72.7	0.02
	pCR	1		8		
Neratinib	No pCR	9	25	6	50.0	0.4
	pCR	3		6		
Trastuzumab plus neratinib	No pCR	4	43	3	62.5	0.6
	pCR	3		5		
All arms	No pCR	20	25.9	12	61.3	0.009
	pCR	7		19		

### 8-Gene trastuzumab benefit predictive signature correlates with pCR

We developed and validated an 8-gene expression signature that predicted trastuzumab benefit in the adjuvant setting in NSABP B-31 [22] and NCCTG9831 [23]. Using RNA-Seq data, the 8-gene signature was used to assign each FB-7 patient to one of the three predicted trastuzumab groups (high, intermediate, and no benefit). In these benefit groups, the pCR rates were 75%, 53%, and 22%, respectively, when analyzed without regard to the treatment arm (Table 5). The difference between the pCR rates of the high (9/12) and no-benefit (2/9) groups was significant (*P* = 0.03). The pCR rate of the combined high and intermediate benefit groups (26/44), compared to the no benefit group (2/9), was higher (59% vs 22%) and showed a trend for significance (*P* = 0.056).

**Table 5:** NSABP FB-7: pathologic complete response (pCR) rates in 8-gene signature trastuzumab benefit groups

8-Gene trastuzumab benefit prediction group	No. of samples	pCR rate in all arms (%)	Trastuzumab	Neratinib	Trastuzumab plus neratinib
No benefit	9	22	1/3 (33%)	0/5 (0%)	1/1 (100%)
Intermediate benefit	32	53	4/8 (50%)	5/10 (50%)	8/14 (57%)
Most benefit	12	75	4/4 (100%)	4/7 (57%)	1/1 (100%)

### RNA-Seq analysis

Whole transcriptome RNA-Seq analysis was used to assess the gene expression profiles in pre-treatment biopsies (*n =* 59 cases) and residual tumors (*n* = 16 cases) (Additional file 1: Methods). As expected, the distribution of intrinsic subtypes in the FB-7 pre-treatment biopsies (*n* = 59) showed that HER2-enriched (61%) was the most common, but other subtypes were also present: basal (11.9%), luminal A (6.8%), luminal B (15.3%), and normal (5%). A similar distribution of subtypes for HER2-enriched, basal, and luminal B were seen in a composite of five different neoadjuvant clinical trials in clinically HER2^+^ patients [24, 25]. The percentage of luminal A was higher in the composite of neoadjuvant trials (24%) compared to FB-7, in which luminal A represented only 6.8% of the patient population.

Intrinsic subtype classifications were assessed for their association with pCR by combining the pre-treatment samples from all three arms. The pCR rates were highest in the basal and HER2-enriched subtypes, 3/5 (60%) and 22/36 (61%), respectively, and lowest in the luminal subtypes 3/9 (33%) as seen elsewhere [24].

Comparison of intrinsic subtypes between the matched pre-treatment biopsies and post-treatment residual tumors revealed that 6/11 (55%) cases converted to a normal-like subtype (Additional file 1: Figure S2). The PAMELA trial [25] similarly observed that nearly half of breast tumors became normal-like (48.9%) after treatment with anti-HER2 therapy.

## Discussion

This study showed a numerical improvement in the pCR rate in all patients treated with trastuzumab plus neratinib (50%) compared to either the trastuzumab arm (38%) or the neratinib arm (33%). This study was not powered to evaluate the statistical significance between the arms but rather to assess the activity in each arm. Similar observations regarding a non-significant, numerical increase in the pCR rate in patients treated with dual anti-HER2 therapy were seen in the CALGB 40601 (*n* = 233) and NSABP B-41 (*n* = 347) trials, both of which included the arms with trastuzumab and the combination of lapatinib with trastuzumab. However, NeoALTTO (*n* = 301), which also tested the combination of trastuzumab plus lapatinib, showed a statistically significant overall increase in the pCR rate for dual anti-HER2 therapy compared to single-agent trastuzumab. The pCR rate in the NeoALTTO trial was lower in the single anti-HER2 arm (29.5%) than that in B-41, CALGB 40601, and NSABP FB-7 (49.4%, 60.1%, and 39.0%, respectively). The low pCR rate of the single-agent arm in NeoALTTO may have contributed to the observed statistically significant increase in the pCR rate in patients treated with dual anti-HER2 therapy.

Among HR^−^ patients, FB-7 was also consistent with four other neoadjuvant trials [11, 12, 17, 26], which showed that dual anti-HER2 therapy (trastuzumab plus pertuzumab or trastuzumab plus TKI) resulted in a numerically higher pCR rate. Among HR^+^ patients, only the NeoALTTO study showed a statistically significant difference between single- and dual anti-HER2 agents (Additional file 1: Table S2). The difference between the pCR rates in HR^+^ (7/23) and HR^−^ (14/19) tumors reached significance in FB-7 patients treated with dual anti-HER2 therapy *P* = 0.01 (Table 3). Taken together, these observations in neoadjuvant trials suggest that HR^−^ patients, more so than HR^+^ patients, may benefit from additional anti-HER therapy beyond trastuzumab.

The results for HR^+^ patients in the neoadjuvant setting were different from those in the extended adjuvant setting. The ExteNET trial [27], which evaluated the benefit of neratinib vs placebo after trastuzumab-based adjuvant therapy in HER2-positive breast cancer, reported statistically significant fewer invasive disease-free survival (iDFS) events in the neratinib group (HR 0.73, 95% CI 0.57–0.92, *P* = 0.0083), which was the most significant in the ER^+^ patients. Inferring results from the observations in the neoadjuvant setting and applying them to the adjuvant or extended adjuvant setting is problematic because of the differences in tumor burden, endpoints, and drug. Although the mechanisms have not been fully elucidated to account for a greater effect of neratinib in HR^+^ patients than in those patients with HR^−^ disease, it has been postulated that there is a bidirectional crosstalk between the estrogen receptor and the HER2 receptor signaling requiring concomitant block in both pathways for therapy efficacy. In the neoadjuvant trials, HR^+^ patients were given hormonal therapy after anti-HER2 therapy, but in ExteNET, most HR^+^ patients received concurrent hormonal therapy. This may partly account for the differential benefit from dual anti-HER2 therapy seen in HR^+^ patients in the neoadjuvant and adjuvant settings. Interestingly, in NSABP B-52 (a neoadjuvant trial in HER2-positive, estrogen receptor-positive women), the concomitant treatment with trastuzumab plus pertuzumab and endocrine therapy did not increase the pCR rate, which may indicate that there is a unique interaction with neratinib and trastuzumab [28].

Using an identical regimen of trastuzumab plus neratinib, the I-SPY2 study reported that neratinib was superior to trastuzumab with a high degree of probability (95%) in HER2^+^/HR^−^ patients as measured by a pCR of 56% in the neratinib group vs 33% in the trastuzumab (control) group. I-SPY2 specifically excluded patients who had HR^+^ and low-risk tumors according to the 70-gene MammaPrint assay. To determine if the FB-7 population was similar to that of I-SPY2, a subset of 45 baseline tissue samples was evaluated by the MammaPrint assay, and only 3 patients (2 patients in arm 1 [trastuzumab] and 1 patient in arm 2 [neratinib]) were found to be low risk, none of whom achieved a pCR. Therefore, it is unlikely that patient eligibility accounts for the discordant results. Another difference in our trial was the randomization procedure. FB-7 used a fixed randomization, whereas I-SPY2 used an adaptive randomization procedure, putting more patients in the treatment arm with a higher rate of pCR.

As expected, the major toxicity associated with neratinib in FB-7 is diarrhea. In the neratinib-alone and trastuzumab plus neratinib arms, nearly all patients experienced some diarrhea, with the occurrence of grade 3 at 31%. During the study, primary prophylactic anti-diarrheal therapy was instituted and the duration of intense anti-diarrheal therapy was increased from 2 to 4 weeks. These measures were somewhat effective in decreasing grade 3 occurrences. Notwithstanding, a dose reduction of neratinib was required in 38% and 52% of patients receiving single-agent neratinib or the combination, respectively. Premature discontinuation of anti-HER2 regimen during the first 16 weeks of treatment occurred in 19%, 21%, and 40% in arms 1, 2, and 3, respectively. Although the addition of neratinib to trastuzumab failed to reach a statistically significant increase in the overall pCR, a sizable proportion of patients did not receive the anticipated full course of therapy. Future development of neratinib will require strategies to improve the management of acute diarrheal toxicities. The CONTROL trial [29] suggests that budesonide, an oral, poorly absorbed steroid, or colestipol, which binds bile acids, may decrease the occurrence of grade 3 neratinib-associated diarrhea and lower the early discontinuation rate. Additional work is required to optimize the dose and schedule of neratinib alone and in combination with other agents. Moreover, the identification of a biomarker to determine whether there is a unique benefit to a TKI beyond anti-HER2 antibodies would be a clinically important advance.

Of the 11 paired tissue samples, 6 (55%) converted to a normal-like subtype in the post-treatment residual tumor sample regardless of the subtype of the matched pre-treatment biopsy. This conversion to a normal-like subtype was not due to a loss of tumor cellularity, because the percent of tumor cells in the residual tumor was similar to the tumor content of the primary biopsy. This conversion of the intrinsic subtype, also observed by others [25], may be due to the selection of normal-like cells that were in the tumor before treatment or due to an actual change of the subtype under the pressure of treatment. Conversion of tumors to a normal-like subtype, if prognostic of recurrence, may be important in determining further treatment of patients with residual disease.

We show that patients with at least one high-affinity *FCGR3A* allele (*FCGR3A-158V*) had a statistically significantly higher pCR rate than patients who were homozygous for the low-affinity allele (*FCGR3A-*158F) across all trastuzumab-based treatment arms. This association was expected because antibody-mediated ADCC activity is higher in patients with *FCGR3A-*158V/V genotypes than in patients with F/F genotypes, probably due in part to the greater affinity of the FCɣRIIIA-158V for the Fc region of an antibody than the FCɣRIIIA-158F [30, 31]. The efficacy of neratinib is not dependent upon ADCC activity, and as predicted, we see no statistically significant differences in the pCR rates between genotypes in the neratinib arm. Although not statistically significant, the addition of neratinib to trastuzumab numerically improved pCR in F/F patients, from 1 in 8 patients to 3 in 7.

We realize that there are several limitations to this study, which prevent firm conclusions in both the clinical findings and the correlative analyses. The small sample size and changing landscape of treatment required several amendments to the study including the addition of a third arm as well as dropping tissue requirements. This necessitated a non-comparative statistical design for the primary endpoint of pCR. As pointed out, there was a trend toward increased pCR in HR^−^ patients with dual anti-HER2 therapy relative to trastuzumab, which is consistent with other similar trials. The correlative findings, although lacking sufficient power to clearly confirm predictive biomarkers, suggest that the HR receptor status, the *FCGR3A-*158V/F SNP, and the 8-gene trastuzumab predictive signature may be useful markers for identifying patients less likely to achieve a pCR.

In agreement with other studies, we show herein that intrinsic subtypes are associated with pCR and may provide predictive value within HER2^+^ disease, but this has not been validated and their clinical utility is somewhat limited [24]. Intrinsic subtypes are not predictive of trastuzumab benefit in the adjuvant setting [32]. In this study, we show that the subset of patients who were *predicted* to receive no benefit from trastuzumab, based on the 8-gene signature in FB-7, had a pCR rate of only 22%. The low pCR rate and the use of a validated signature indicating that they are unlikely to receive trastuzumab benefit suggest that this patient population would constitute a subset of patients who might benefit from additional or alternative therapies. Such an approach may be useful in the design of new clinical trials following validation of the 8-gene signature in the neoadjuvant setting. Ultimately, the goal of studying novel therapies in the neoadjuvant setting is to personalize treatment to optimize benefit and minimize potential toxicities.

## Conclusions

Overall, trastuzumab plus neratinib resulted in a numerical improvement in the pCR rate (50%) compared to either the trastuzumab arm (39%) or the neratinib arm (33%), with pCR for each arm being greater in the HR^−^ patients than HR^+^ patients (Table 3). However, in spite of primary anti-diarrheal prophylaxis, diarrhea remains the major toxicity associated with neratinib and requires further investigation for mitigation.

Across all trastuzumab-based treatments, patients with at least one high-affinity *FCGR3A* allele (*FCGR3A-158V*) had a statistically significantly higher pCR rate than patients who were homozygous for the low-affinity allele (*FCGR3A-*158F). Neratinib, which is not dependent upon ADCC activity, showed no statistically significant differences in the pCR rates between genotypes. Finally, we show that our 8-gene signature was predictive of pCR and warrants further investigation in HER2-positive patients.

## Supplementary information

**Additional file 1: Methods. Table S1.** Paclitaxel, trastuzumab, and neratinib drug intensity during weekly paclitaxel phase: NSABP FB-7. **Table S2.** Pathologic complete response (pCR) rates in neoadjuvant HER2^+^ breast cancer trials: NSABP FB-7. **Figure S1.** MammaPrint Scores for patients enrolled in NSABP FB-7. Randomized patients were assigned a MammaPrint score based on the propriatory test from Agendia. **Figure S2.** Intrinsic subtypes differ in residual tumors compared to the pretreatment biopsy: NSABP FB-7.

## Data Availability

The datasets during and/or analyzed during the current study will be available from the corresponding author on reasonable request and with permission of PUMA Biotechnology. Name of the registry: ClinicalTrials.gov Trial registration number: NCT01008150 Date of registration: November 3, 2009
