# Correction to: NSABP FB-7: a phase II randomized neoadjuvant trial with paclitaxel + trastuzumab and/or neratinib followed by chemotherapy and postoperative trastuzumab in HER2+ breast cancer

**DOI:** 10.1186/s13058-019-1240-y

**Published:** 2020-01-22

**Authors:** Samuel A. Jacobs, André Robidoux, Jame Abraham, José Manuel Pérez-Garcia, Nicla La Verde, James M. Orcutt, Marina E. Cazzaniga, Fanny Piette, Silvia Antolín, Elena Aguirre, Javier Cortes, Antonio Llombart-Cussac, Serena Di Cosimo, Rim S. Kim, Huichen Feng, Corey Lipchik, Peter C. Lucas, Ashok Srinivasan, Ying Wang, Nan Song, Patrick G. Gavin, April D. Balousek, Soonmyung Paik, Carmen J. Allegra, Norman Wolmark, Katherine L. Pogue-Geile

**Affiliations:** 1 NSABP Foundation, Inc., Nova Tower 2, Two Allegheny Center – Ste 1200, Pittsburgh, PA 15212 USA; 2 Centre hospitalier de l’université de Montréal, Montréal, QC Canada; 3 Cleveland Clinic, Cleveland, OH USA; 4 QuironSalud Group, IOB Institute of Oncology, Barcelona, Madrid Spain; 5 Medica Scientia Innovation Research (MedSIR), Barcelona, Spain; 6 Present address: ASST Fatebenefratelli Sacco - PO Luigi Sacco, Milan, Italy; 7 ASST Fatebenefratelli Sacco - PO Fatebenefratelli, Milan, Italy; 8 Roper St. Francis Healthcare, Charleston, SC USA; 9 Azienda Ospedaliera San Gerardo, Monza, Italy; 10 International Drug Development Institute (IDDI), Louvain-la-Neuve, Belgium; 11 Hospital Universitario, Coruña, Spain; 12 Fondazione IRCCS Istituto Nazionale di Tumori, Milan, Italy; 13 Department of Pathology, University of Pittsburgh School of Medicine, Pittsburgh, PA USA; 14 Severance Biomedical Science Institute and Department of Medical Oncology, Yonsei University College of Medicine, Seoul, Republic of South Korea; 15 Department of Medicine, University of Florida Health, Gainsville, FL USA; 16 University of Pittsburgh, Pittsburgh, PA 15212 USA

**Correction to: Breast Cancer Res**

**https://doi.org/10.1186/s13058-019-1196-y**

After the publication [1] the authors report corrections for Table 3, for the headers of columns 5 and 7, which should read as follows: Column 5 Header: *P*-value for pCR, T vs Other Arms in HR^+^, and Column 7 Header: *P*-value for pCR, T vs Other Arms in HR^–^.

The correct version of the table can be found below:

**Table 3:** NSABP FB-7: Pathologic complete response (pCR) (breast and nodes) by treatment arm and HR status

Treatment arms, *n* = 42 each arm	Total pCR	*P-value* for pCR T vs Other Arms in all patients^a^	pCR HR^+^	*P-value* for pCR T vs Other Arms in HR^+ b^	pCR HR^−^	*P-value* for pCR T vs Other Arms in HR^− b^	*P-value* HR^+^ vs HR^−^
Arm 1 (T)	16/41 (39.0%)	NA	8/27 (29.6%)	NA	8/14 (57.1%)	NA	0.11
Arm 2 (N)	14/42 (33.3%)	0.63	8/29 (27.6%)	1	6/13 (46.2%)	0.71	0.30
Arm 3 (T plus N)	21/42 (50%)	0.22	7/23 (30.4%)	1	14/19 (73.7%)	0.46	0.01
All arms	51/126 (40%)		23/79 (29%)		28/46 (61%)		0.001

Abbreviations: T, trastuzumab; N, neratinib

^a^CMH test stratified for the time period the patients were randomized to the study (prior/after the addition of Arm 3)

^b^Fisher’s exact test

## References

[1] Jacobs (2019). Breast Cancer Res.

