# Supplementary material for: NSABP FB-7: a phase II randomized neoadjuvant trial with paclitaxel + trastuzumab and/or neratinib followed by chemotherapy and postoperative trastuzumab in HER2+ breast cancer
Source: Breast Cancer Res. 2019 Dec 3;21:133. doi: 10.1186/s13058-019-1196-y (PMC6892191; doi:10.1186/s13058-019-1196-y)
Supplement: Supplementary file 1 — Additional file 1: Methods. Table S1. Paclitaxel, trastuzumab, and neratinib drug intensity during weekly paclitaxel phase: NSABP FB-7. Table S2. Pathologic complete response (pCR) rates in neoadjuvant HER2+ breast cancer trials: NSABP FB-7. Figure S1. MammaPrint Scores for patients enrolled in NSABP FB-7. Randomized patients were assigned a MammaPrint score based on the propriatory test from Agendia. Figure S2. Intrinsic subtypes differ in residual tumors compared to the pretreatment biopsy: NSABP FB-7. [file 13058_2019_1196_MOESM1_ESM.docx]

**Additional file 1**

**A Phase II Randomized Neoadjuvant Trial with Paclitaxel plus Trastuzumab**

**and/or Neratinib with Postoperative Chemotherapy plus Trastuzumab**

**in HER2^+^ Breast Cancer: NSABP FB-7**

**Table of Contents**

**Supplementary Methods pg 2**

**Supplementary Tables**

**Table S1: Paclitaxel, trastuzumab, and neratinib drug intensity**

**during weekly paclitaxel phase: NSABP FB-7 pg 3**

**Table S2: Pathologic complete response (pCR) rates in neoadjuvant**

**HER2^+^ breast cancer trials: NSABP FB-7 pg 4**

**Supplementary Figures**

**Figure S1:** **MammaPrint Scores for patients enrolled in NSABP FB-7**

Randomized patients were assigned a MammaPrint score based on the

propriatory test from Agendia **pg 5**

**Figure S2: Intrinsic subtypes differ in residual tumors compared**

**to the pretreatment biopsy: NSABP FB-7 pg 6**

**Supplementary Methods**

**RNA-Seq:** Starting material for library construction was 100 ng RNA. Ribosomal depletion was carried out using the Epicentre Ribo-Zero™ rRNA Removal Kit (Human/Mouse/Rat). The recommended Ribo-Zero reaction setup for 1 µg starting material was reduced by 75% for our 100 ng input with no discernable effect on removal efficiency. Cleanup of the ribosomal-depleted RNA was performed with Beckman Coulter Agencourt RNAClean XP magnetic beads.

RNA-Seq libraries were prepared by amplifying and converting the RNA into cDNA with the SeqPlex RNA Amplification Kit for whole genome transcriptome amplification from Sigma-Aldrich. The cDNA libraries were constructed with a Life Technologies kit for the preparation of genomic DNA sequencing libraries following the manufacturer's recommendations but fragmentation of the cDNA was not performed because the amplified cDNA was already of appropriate size for library preparation. Eight barcoded libraries were quantitated and pooled, and the Ion Chef system was used to prepare templates and load chips. Sequencing was performed on the Ion Proton system.

**Table S1:** Paclitaxel, trastuzumab, and neratinib drug intensity during weekly paclitaxel phase: NSABP FB-7

| **Treatment** | **Arm 1** | **Arm 2** | **Arm 3** |
| --- | --- | --- | --- |
| Paclitaxel mg/m2/week; % of target | 94% | 90% | 84% |
| Neratinib mg/day; % of target | − | 88% | 84% |
| Trastuzumab mg/kg/week; % of target | 105% | − | 95% |
| Number of patients with at least 1 dose reduction: |  |  |  |
| Paclitaxel | 10% | 12% | 12% |
| Neratinib | − | 38% | 52% |
| Discontinued before completion of 4 cycles | 19% | 21% | 40% |

**Table S2:** Pathologic complete response (pCR) rates in neoadjuvant HER2^+^ breast cancer trials: NSABP FB-7

| **Study** | **HR^+^ pCR** | **HR^+^ pCR** | **HR^+^** | **HR^-^ pCR** | **HR^-^ pCR** | **HR^-^** | **Reference** |
| --- | --- | --- | --- | --- | --- | --- | --- |
|  | **Single anti-HER2 (Trast)** | **Dual anti-HER2** | ***P*** | **Single anti-HER2 (Trast)** | **Dual anti-HER2** | ***P*** |  |
| NeoSphere | 10/50 (20%) | 13/50 (26%) | 0.64 | 21/57 (36.8%) | 36/57 (63.2%) | 0.009 | Gianni  *Lancet Oncol* 13:25,2012  (24) |
| NeoALTTO | 17/75 (22.7%) | 32/77 (41.6%) | 0.020 | 27/74 (36.5%) | 46/75 (61.3%) | 0.004 | Baselga  *Lancet* 379:633,2012 (11) |
| NSABP  B-41 | 55/121 (45.5%) | 59/108 (54.6%) | 0.21 | 32/55 (58.2%) | 44/63 (69.8%) | 0.26 | Robidoux  *Lancet Oncol* 14:1183,2013  (25) |
| CALGB 40601 | 27/69 (39%) | 28/69 (41%) | 1 | 24/50 (54%) | 32/47 (68%) | 0.073 | Carey  *J Clin Oncol*  34:542,2016  (12) |
| NSABP  FB-7 | 8/27 (29.6%) | 7/23 (30.4%) | 1 | 8/14 (57.1%) | 14/19 (73.7%) | 0.46 | Current study |

**Figure S1:** MammaPrint scores for patients enrolled in NSABP FB-7


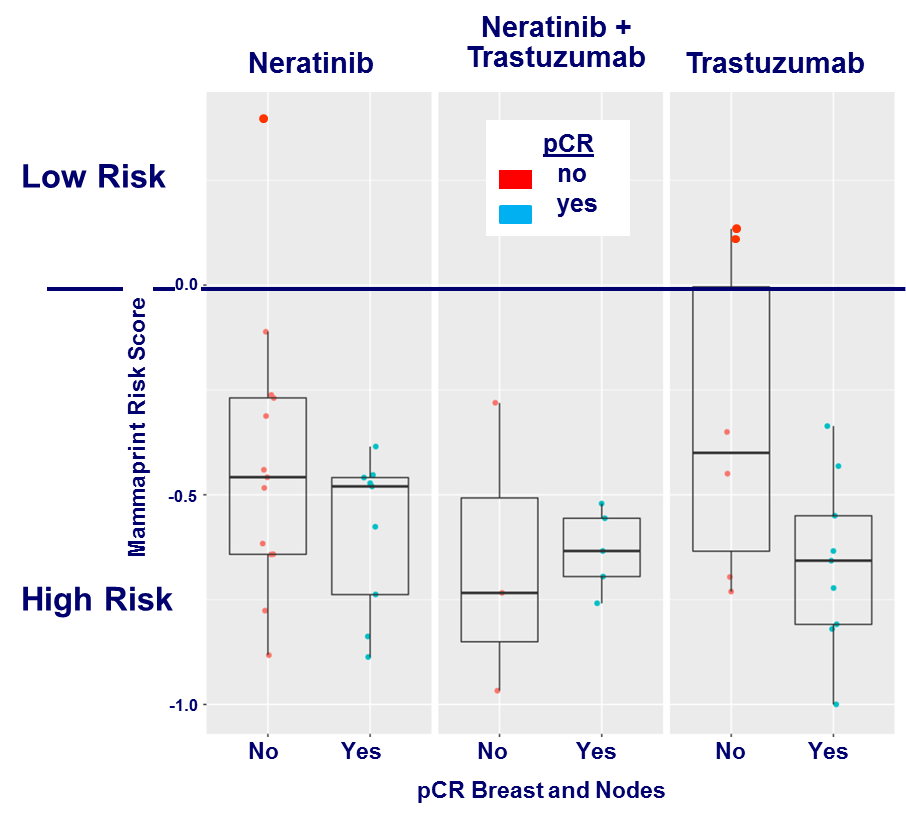


Randomized patients were assigned a MammaPrint score based on the propriatory test from Agendia.

**Figure S2:** Intrinsic subtypes differ in residual tumors compared to the pretreatment biopsy NSABP FB-7

**
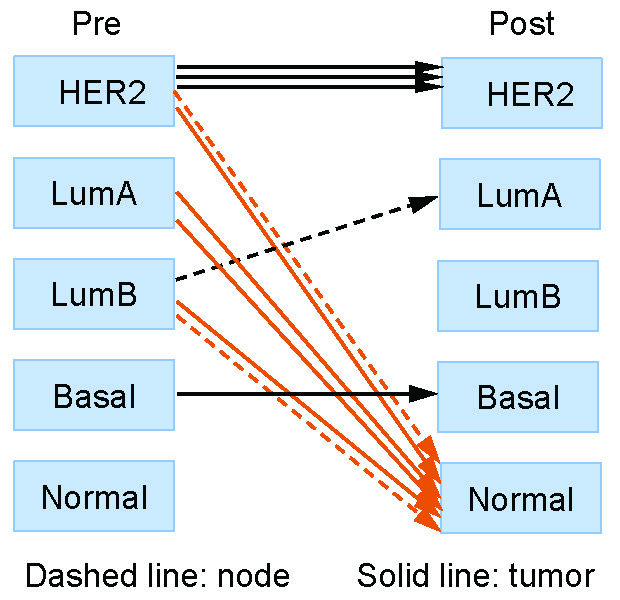
**

To identify breast cancer subtypes, RNA-Seq data was used to subtype pre-treatment and post-treatment residual tumors using the AIMS method with RPKM normalization. The AIMS method tends to be more robust than other methods such as PAM50 because the gene-centering step in PAM50 is highly dependent on the composition of other tumors in a reference set. Subtypes of matched pre-treatment biopsies were compared to post-treatment residual tumors. In residual breast tissue, 4/8 (50%) of the residual tumors were normal-like even though the matched pre-treatment biopsy tumors were HER2-enriched (*n*=1), luminal A (*n*=2), or luminal B (*n*=1). The subtypes of four other patients were the same in the pre-treatment and residual tumors (3 HER2-enriched and 1 basal). In three other cases, the post-treatment tumor samples were from lymph nodes including two with a luminal B subtype and one that was HER2-enriched. In two cases the subtype of the residual disease was normal-like and in one the luminal B subtype converted to a luminal A subtype. Importantly, for residual disease subtyped as normal-like, we confirmed that the tumor cellularity was ≥50% based on examination of an H&E slide. In this limited sample set, a noteworthy rate of conversion to the normal-like subtype following HER2-targeted therapy was observed in residual disease (6/11 [55%]).
